# Prognostic Value of Human Apurinic/Apyrimidinic Endonuclease 1 (APE1) Expression in Breast Cancer

**DOI:** 10.1371/journal.pone.0099528

**Published:** 2014-06-10

**Authors:** Joohyun Woo, Heejung Park, Sun Hee Sung, Byung-In Moon, Hyunsuk Suh, Woosung Lim

**Affiliations:** 1 Department of Surgery, Ewha Womans University School of Medicine, Ewha Womans University Mokdong Hospital, Seoul, Korea; 2 Department of Pathology, Ewha Womans University School of Medicine, Ewha Womans University Mokdong Hospital, Seoul, Korea; 3 Department of Plastic surgery, Ewha Womans University School of Medicine, Ewha Womans University Mokdong Hospital, Seoul, Korea; Baylor College of Medicine, United States of America

## Abstract

Human apurinic/apyrimidinic endonuclease 1 (APE1) is an essential protein for DNA base excision repair (BER) and redox regulation. The ability of cancer cells to recognize DNA damage and initiate DNA repair is an important mechanism for therapeutic resistance. Several recent studies have suggested that APE1 expression levels and/or subcellular dysregulation may be used to indicate the sensitivity of tumors to radiotherapy or chemotherapy. In this study, we assessed the prognostic significance of APE1 and differences in APE1 expression levels according to breast cancer molecular subtypes. We analyzed formalin-fixed, paraffin-embedded tumor tissue sections from 243 cases diagnosed as invasive breast cancer at Ewha Womans University Medical Center between January 2003 and December 2008. Immunohistochemistry was performed and the nuclear level of APE1 was scored by taking into account the percentage of positive cells. Medical records were reviewed to investigate clinicopathologic characteristics. We found that nuclear APE1 high-level expression (proportion ≥50%) in breast cancer showed a tendency towards unfavorable prognosis regarding disease-free survival (p = 0.093). However, there was no significant difference in overall survival between low and high-level expression groups (p = 0.294). Interestingly, within the Ki-67 low-level expression group, APE1 low-level expression was significantly associated with poor overall survival (p = 0.007). A significant positive correlation was observed between APE1 nuclear expression and estrogen receptor status (75.7% vs. 59.7%, p = 0.022). Also, the luminal A subtype was the most commonly observed breast cancer subtype in the APE1 high-level expression group (61.6% vs. 45.2%, p = 0.000). This study suggests that APE1 expression may be associated with breast cancer prognosis. In particular, its role as a prognostic factor would be significant for breast cancers with a low Ki-67 proliferation index. It is proposed that nuclear APE1 may be a novel target in breast cancer with a low proliferation rate to obtain better outcome.

## Introduction

Human apurinic/apyrimidinic endonuclease 1 (APE1, also called HAP1) is a multi-functional protein involved in DNA repair and redox regulation. APE1 is a class II apurinic apyrimidinic endonuclease that incises DNA to cause a nick in the backbone creating an AP-site, which acts as a recognition site for enzymes subsequently involved in the base excision repair (BER) pathway [1]. An alternative name for APE1 is redox effector factor-1 (Ref-1), because this protein was also identified as a redox modulator of transcription factors (TFs) including Fos, Jun, nuclear factor-κB (NFκB), HIF-1α and p53 [2]. In addition to these activities, APE1 has specific roles in regulating cell fate and is involved in the control of different cellular process such as apoptosis, proliferation and differentiation [3]. The human APE1 gene (∼2.6 kb in size) is localized on chromosome 14 q11.2-12 and consists of four introns and five exons [4], [5]. APE1 is a globular α/β protein that possesses both DNA repair and redox regulatory activities. The N-terminal domain is essential for the redox activity of APE1 while the C-terminus is essential for DNA repair activity [6].

The distribution of APE1 in many cell populations has been reported to be varied and complex. Though most studies showed that APE1 was localized to the nucleus, in some cell types APE1 displayed only cytoplasmic expression or both nuclear and cytoplasmic localization [3]. The biological relevance of the distinct subcellular localization of APE1 is not clearly understood.

There is accumulating evidence that altered APE1 expression patterns are associated with carcinogen susceptibility and cancer development or progression. Overexpression or an atypical subcellular distribution pattern of APE1 has been observed in breast cancer, non-small cell lung cancer, head and neck cancer, osteosarcomas, germ cell tumors and ovarian cancer [7]–[12]. These studies suggested that APE1 could be associated with survival outcome, lymph node status, proliferation index and resistance to chemotherapy or radiotherapy.

Estrogen receptor α (ERα) is a ligand-inducible transcription factor that plays a critical role in carcinogenesis and tumor progression of breast cancer. ERα has a modular structure and region C contains the DNA-binding domain composed of two zinc finger motifs, the second zinc finger being particularly vulnerable to oxidative stress [13]. Oxidation of ERα precludes the ability of the receptor to interact with DNA and alters estrogen-responsive gene expression [14], [15].

A recent study showed that endogenously expressed APE1 and ERα in MCF-7 human breast cancer cells interact and that APE1 enhances the interaction of ER with estrogen response elements (EREs) in DNA. It has also been demonstrated that the redox activity of APE1 is instrumental in altering estrogen-responsive gene expression [16].

In the present study, we assessed the prognostic significance of APE1 and the association of APE1 expression levels with ER in breast cancer to explore the pattern of APE1 expression in terms of clinical and biological factors.

## Materials and Methods

### Ethics statement

The Institutional Review Board of the Ewha Clinical Trial Center at Ewha Womans University Medical Center, Korea approved this study protocol and written informed consent was obtained from all patients.

### Patients and surgical specimens

We analyzed retrospectively formalin-fixed, paraffin-embedded normal breast tissue sections from 30 cases and tumor tissue sections from 243 cases of stages I, II and III primary invasive breast cancer diagnosed at Ewha Womans University Medical Center between January 2003 and December 2008. Samples of breast tissue were obtained from surgical specimens following breast-conserving surgery or modified radical mastectomy without neoadjuvant chemotherapy. The medical records were reviewed for clinicopathologic characteristics and follow-up data for all patients were obtained with a median follow-up period of 61 months. Patients who had been diagnosed with other malignancies before surgery or during the follow-up period were excluded, except for those with nonmelanomatous skin cancer, cervical intraepithelial neoplasms or papillary carcinoma of the thyroid. Cases in which human epidermal growth factor receptor 2 (HER2) expression was equivocal (2+) using immunohistochemistry (IHC) were also excluded.

### IHC staining of APE1

Formalin-fixed, paraffin-embedded sections (4 µm) of tumor tissues were deparaffinized in xylene and rehydrated in xylene and graded alcohols. For antigen retrieval, slides were placed in a target retrieval solution (pH 6.0, DAKO) and boiled for 10 min in a microwave oven. Slides were then rinsed with distilled water for 10 min, followed by TBST buffer (tris-buffered saline +0.1% Tween 20) for 5 min, repeated twice. After quenching of the endogenous peroxidase-blocking agent for 10 min, slides were rinsed twice with TBST buffer for 5 min.

Following application of protein-blocking serum for 10 min, tissue sections were incubated in a humidified box at 4°C overnight with the primary antibody (1∶100 dilution; Novus Biologicals, Littleton, CO, USA) without washing. The next day, sections were treated with the EnVision+ System-HRP secondary antibody (Dako) for 30 min. Finally, all sections were visualized using chromogen (AEC+ High Sensitivity Substrate, Dako) and counterstained with hematoxylin (Dako).

To evaluate the expression of APE1, the mean number of cells per field in five nonoverlapping intratumoral fields (breast cancer) or in five random hot spots (normal breast) was counted under high-power magnification (×200). An experienced pathologist scored the percentage of positive cells and IHC staining intensity in paraffin sections according to the scoring system suggested by Remmele and Stegner [17].

### Evaluation of ER, progesterone receptor (PR), HER2 status and Ki-67

ER, PR and HER2 status in tumors obtained from core needle biopsy samples before surgery or from surgical specimens was determined using IHC according to the Allred scoring system and the FDA-approved scoring system for HER2. Cutoff values for ER, PR and HER2 were 2%, 2% and 30%, respectively. The Ki-67 index was determined by counting the number of positive cells with nuclear staining in at least 500 tumor cells under high magnification (×400) and was expressed as a percentage of the total positive cells. For statistical analysis, based on a previous study [18], we used 14% as a cutoff point. Breast cancer molecular subtypes were defined according to the 2011 St. Gallen consensus [19].

### Statistics

SPSS software version 20 (SPSS, Chicago, IL, USA) was used for all statistical analyses. The association between clinicopathologic parameters and APE1 expression was analyzed using the Chi-square test. Overall survival (OS) and disease-free survival (DFS) rates were assessed using the Kaplan-Meier method. Statistical significance between life tables was determined with the log-rank test and Breslow test. Significant associations between prognostic factors and survival rates within each subgroup were evaluated using univariate Cox regression analysis. Furthermore, multivariate Cox regression analysis was used to determine the independent prognostic value within the stratified cohorts. A p-value of less than 0.05 was considered statistically significant.

## Results

### APE1 expression in normal breast and invasive breast cancer

**Figure 1:**
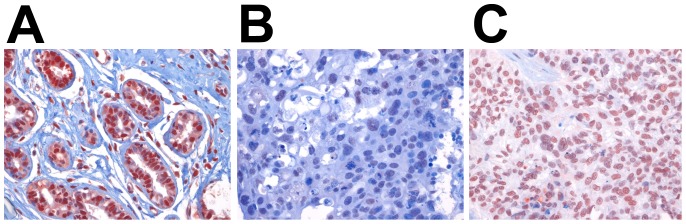
Immunohistochemical staining of APE1. **A**. Nuclear staining in the luminal epithelium of normal breast ducts and lobules. **B**. Low, and **C**. high nuclear APE1 expression in invasive breast cancer. (magnification ×400).

IHC, used to evaluate APE1 expression, revealed that staining intensity of the APE1 protein was strong in all 30 normal breast tissues. All normal breast tissues consistently showed nuclear staining in the luminal epithelium of breast ducts and lobules (Figure 1 A). The mean proportion of positive cells was 70–90%. There was no case with cytoplasmic or mixed staining in normal breast.

A similar pattern was observed for breast cancers. Moderate to strong nuclear staining was seen in the epithelia of 239 (98.4%) breast cancer tissues. Only four out of 243 cases (1.6%) displayed cytoplasmic staining. It appeared that the intensity or subcellular location of APE1 did not correlate with clinicopathologic parameters. However, the proportion of positive tumor cells varied among samples. Therefore, analysis of IHC data was performed by scoring the percentage of positive tumor cells with nuclear staining. The APE1 low-level expression group was defined as breast cancer paraffin sections with <50% of tumor cells with positive immunostaining (Figure 1 B), and the APE1 high-level expression group was defined as those with ≥50% of tumor cells with positive immunostaining (Figure 1 C), which we have used in previous studies.

### APE1 expression and clinicopathologic correlation

The number of patients in the APE1 low-level expression group was 62 (25.9%) and that in the APE1 high-level expression group was 177 (74.1%). Clinicopathologic characteristics of each group according to APE1 expression levels are summarized in Table 1. Age of patients, tumor size, lymph node metastatic status, histologic grade and HER2 status did not correlate with APE1 expression (Table 1). ER and PR status were positively correlated with APE1 high-level expression (p = 0.022, 0.042). Regarding molecular subtypes, the luminal A subtype was observed more frequently in the APE1 high-level expression group, while the basal-like subtype was significantly associated with the APE1 low-level expression group (p = 0.000).

**Table 1:** Relationship between APE1 expression and clinicopathological parameters.

Characteristic		APE1 low-level	APE1 high-level	
		expression (n = 62)	expression (n = 177)	
		No. of patients (%)	No. of patients (%)	p-value
**Age (years)**				0.301
	<50	30 (48.4)	100 (76.9)	
	≥50	32 (51.6)	77 (43.5)	
**T stage**				0.159
	T1-T2	57 (91.9)	170 (96.6)	
	T3-T4	5 (8.1)	6 (3.4)	
**Lymph node metastasis**				0.448
	Negative	42 (67.7)	110 (62.1)	
	Positive	20 (32.3)	67 (37.9)	
**Estrogen receptor**				0.022
	Negative	25 (40.3)	43 (24.3)	
	Positive	37 (59.7)	134 (75.7)	
**Progesterone receptor**				0.042
	Negative	28 (45.2)	53 (29.9)	
	Positive	34 (54.8)	124 (70.1)	
**HER2**				0.751
	Negative	44 (71.0)	120 (67.8)	
	Positive	18 (29.0)	57 (32.2)	
**Histologic grade**				0.263
	I	24 (38.7)	56 (31.6)	
	II	22 (35.5)	84 (47.5)	
	III	16 (25.8)	37 (20.9)	
**Lymphovascular invasion**				0.366
	Yes	47 (75.8)	118 (66.7)	
	No	15 (24.2)	58 (32.8)	
	Uncertain	0 (0)	1 (0.6)	
**Ki-67**				
	<14%	37 (59.7)	114 (64.4)	0.506
	≥14%	25 (40.3)	63 (35.6)	
**Molecular subtype**				0.000
	Luminal A	28 (45.2)	109 (61.6)	
	Luminal B	10 (16.1)	30 (16.9)	
	HER2	7 (11.3)	26 (6.8)	
	Basal-like	17 (27.4)	12 (6.7)	

### Relationship of APE1 expression to disease prognosis

**Figure 2:**
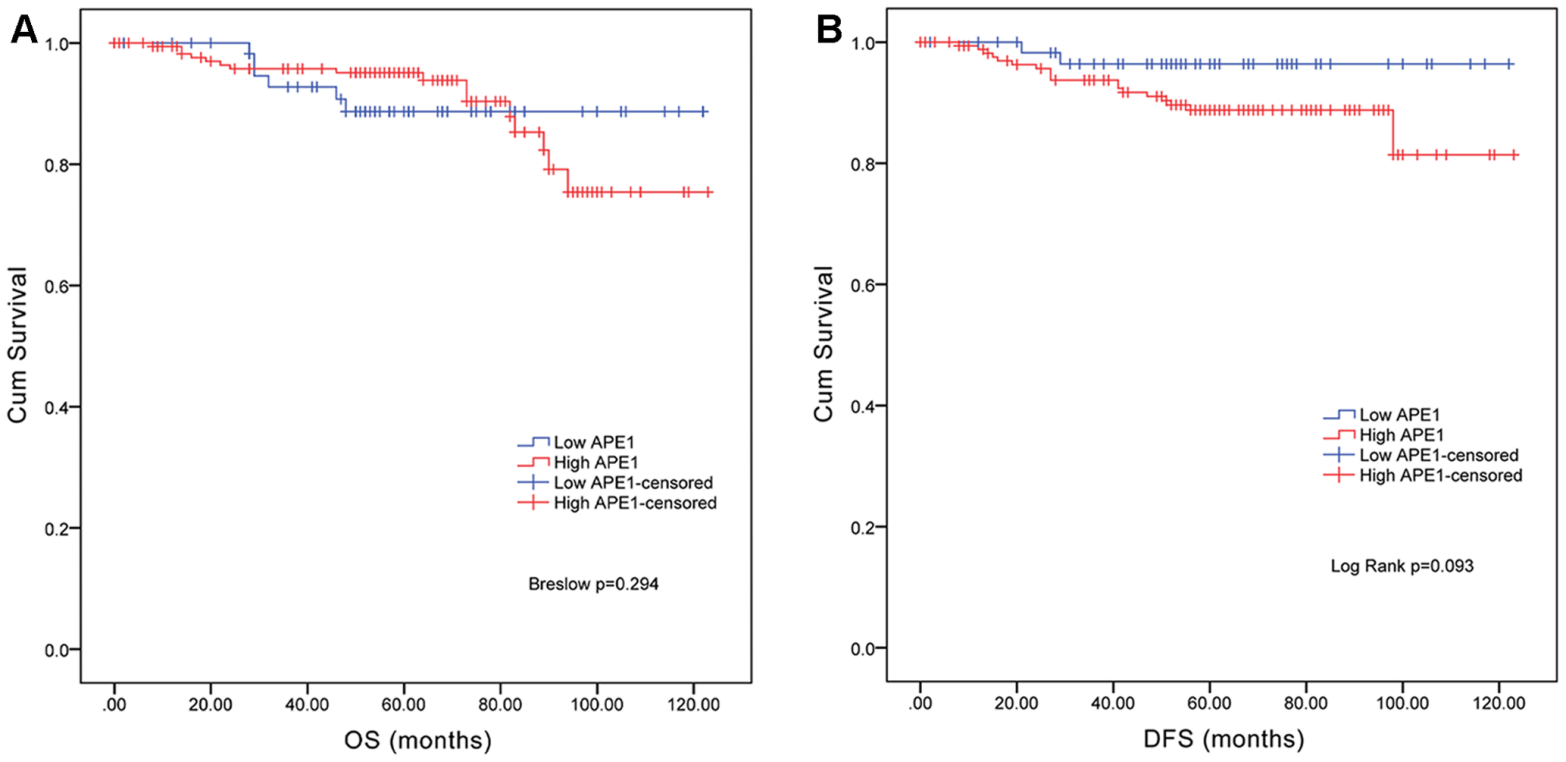
Kaplan-Meier survival curves based on APE1 expression levels. **A**. Overall survival (OS). **B**. DFS. There was no significant difference in OS rates between the APE1 high-level expression group and the APE1 low-level expression group. There was a difference in DFS rates; patients in the APE1 low-level expression group showed a tendency to relapse. (High APE1 (*red*), low APE1 (*blue*).)

**Figure 3:**
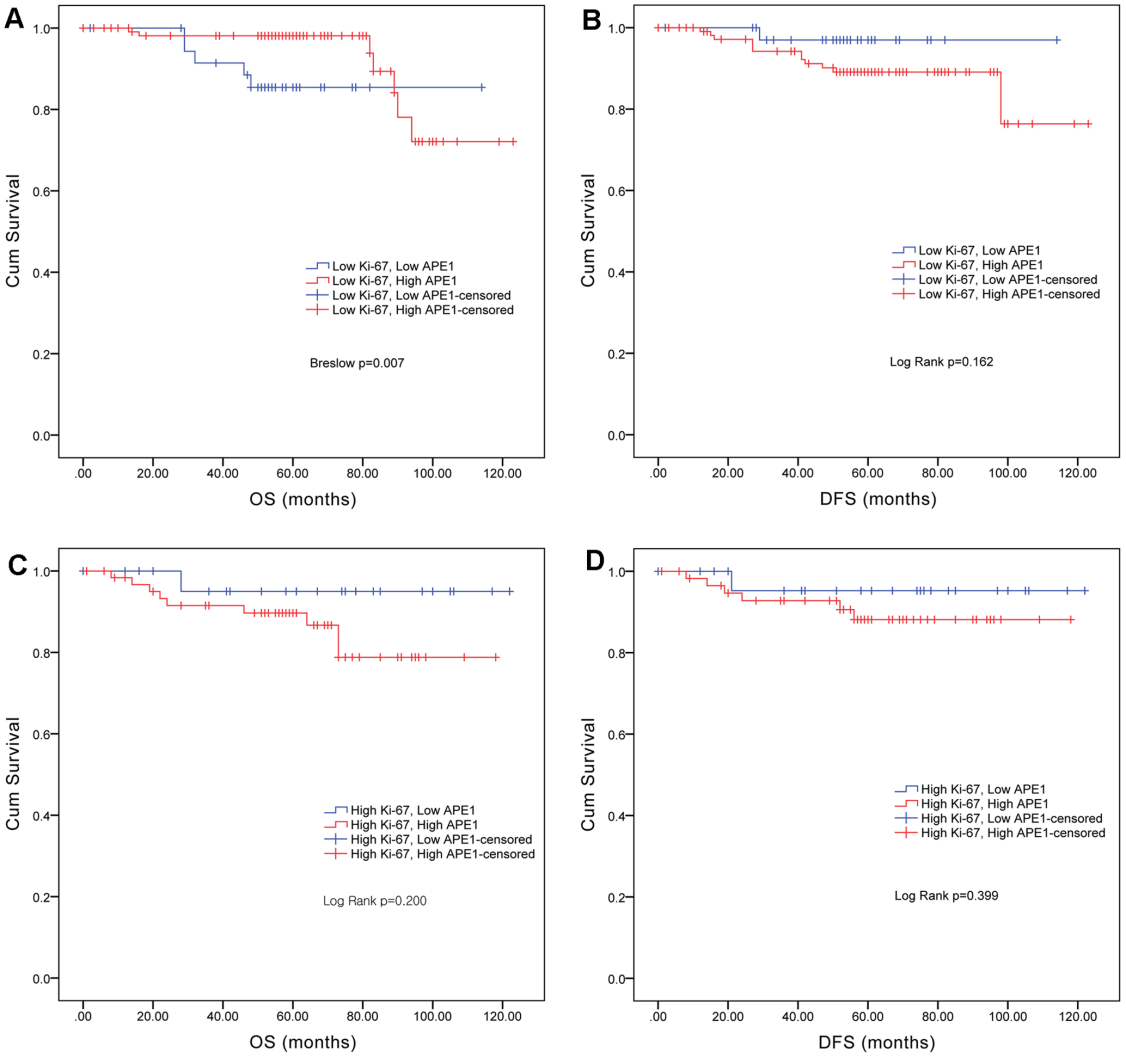
Kaplan-Meier survival curves based on APE1 expression levels in Ki-67-stratified cohorts. **A**. OS in the Ki-67 low-level expression cohort. **B**. DFS in the Ki-67 low-level expression cohort. **C**. OS in the Ki-67 high-level expression cohort. **D**. DFS in the Ki-67 low-level expression cohort. Within the Ki-67 low-level expression group, APE1 low-level expression was associated with poor OS and within the Ki-67 high-level expression group, APE1 low-level expression was associated with good OS. (High APE1 (*red*), Low APE1 (*blue*).)

To evaluate APE1 expression as a prognostic factor, OS and DFS rates were obtained. The Breslow test (generalized Wilcoxon) showed that there was no significant difference in OS rates between the APE1 low-level expression group and APE1 high-level expression group (p = 0.294, Figure 2). With respect to differences in DFS rates using the log-rank test, patients in the APE1 low-level expression group had a tendency to relapse (p = 0.092, Figure 2). Interestingly, survival curves using the Kaplan-Meier method revealed that within the Ki-67 low-level expression group, APE1 low-level expression was associated with poor OS, and within the Ki-67 high-level expression group, APE1 low-level expression was associated with good OS. Univariate Cox regression analysis showed that APE1 expression was a significant prognostic factor in Ki-67 low-level but not high-level expression cases (p = 0.007, Figure 3). Also, multivariate Cox regression subgroup analysis revealed that among Ki-67 low-level expression cases, there was a significant association between APE1 expression and the OS rate (p = 0.045, Table 2).

**Table 2:** Cox regression analysis of nuclear APE1 expression on OS in invasive breast cancer defined by the Ki-67 low-level expression cohort.

Characteristic		OS (n = 151)			
		n	HR	95% CI	p-value
**Age (years)**				0.174–2.516	0.543
	<50	72	1		
	≥50	65	0.661		
**T stage**				0.824–21.996	0.084
	T1-T2	130	1		
	T3-T4	7	4.258		
**Lymph node metastasis**				0.196–2.876	0.675
	Negative	92	1		
	Positive	45	0.750		
**Estrogen receptor**				0.262–25.453	0.416
	Negative	20	1		
	Positive	117	2.584		
**HER2**				0.314–5.272	0.726
	Negative	105	1		
	Positive	32	1.286		
**Histologic grade**				0.302–9.010	0.564
	I+II	122	1		
	III	15	1.649		
**APE1 expression**				0.039–0.962	0.045
	Low	32	1		
	High	105	0.193		

## Discussion

Different human cell types have exhibited differential APE1 localization. Existing reports show that although lactating glandular epithelial cells of the breast showed cytoplasmic localization, normal epithelial cells displayed a mainly nuclear localization, and normal cells of the liver and ovary also exhibited predominantly nuclear localization [20]–[22]. In normal lung tissues, a mixed pattern of both nuclear and cytoplasmic localization was observed [8]. Though levels or patterns of APE1 expression in human tumors were also varied, as described in previous studies, it was obvious that these changed in tumor cells and were different from those in normal cells.

In the current study, the predominant pattern of APE1 expression was nuclear both in normal breast and in breast cancer; however, the level of nuclear APE1 expression was altered in breast cancer. Similarly, Puglisi *et al*. showed that APE1 expression was predominantly localized in the nucleus of breast tumor cells [7], [9]–[11], [23]. It was reported by Kakolyris *et al*. that both nuclear and cytoplasmic expressions were common in breast carcinomas [20]–[22].

We demonstrated that APE1 high-level expression was positively correlated with ER and PR expression using semi-quantitative IHC. In a previous study, Carol *et al*. showed that endogenously expressed ER interacted with APE1 in a breast cancer cell line and that APE1 also enhanced the binding of ERα to ERE-containing DNA, suggesting that APE1 acted to maintain the reduced and active forms of ERα. The results suggested that the redox activity of APE1, but not its DNA repair activity, was involved in mediating these effects [16]. Furthermore, because APE1 had the ability to foster repair of estrogen-responsive genes, it might be involved in facilitating stabilization of ERα-ERE complex formation and maintaining genomic integrity [23].

This significant relationship between APE1 and ER activity *in vitro* was observed in the present study. Additionally, statistical analysis revealed that there was a significant correlation between APE1 high-level expression and the luminal A breast cancer subtype, even though HER2 status or the Ki-67 proliferative index was not associated with APE1 expression levels.

Although high levels of nuclear APE1 expression were more common in the luminal A breast cancer subtype that had a better prognosis than other breast cancer subtypes, high APE1 expression might be correlated with poor OS and DFS rates. However, the difference in OS rates in patients with high or low-level APE1 tumor expression was not statistically significant and APE1 high-level expression had a tendency to be associated with reduced DFS time.

Most studies to determine whether APE1 can be used as a predictive marker showed that high-level expression or cytoplasmic staining was associated with poor outcome and resistance to chemoradiotherapy in patients with lung cancer, breast cancer, head and neck cancer, osteosarcomas, germ cell tumors and hepatocellular carcinomas [7], [9]–[11], [24]. On the other hand, nuclear staining correlated with a low proliferation index, p53-negative and survival benefit in non-small cell lung cancer, and with decreased angiogenesis and negative lymph nodes in breast cancer in other studies [8], [24].

Nuclear localization of APE1 is thought to indicate that it functions in DNA repair while its cytoplasmic localization has been associated with its role in mitochondrial DNA repair or redox regulation of transcription factors [3]. In a state of oxidative stress, such as malignancy, APE1 expression is induced in response to increased radical oxygen species (ROS), and dysregulation of APE1 allows cancer cells to continue to survive. In addition, as a consequence of the increased APE1 activity that accompanies tumorigenesis, resistance to cytotoxic agents such as alkylating agents and platinum compounds and to ionizing radiation used in the treatment of tumors to induce DNA damage in cells could be enhanced [25].

In the current study we could not find strong evidence to support the use of APE1 as a prognostic factor, although analysis of subgroups stratified by parameters that would be associated with APE1 expression in the present study or previous studies showed some interesting results. For each subgroup stratified by ER, PR, molecular subtype, p53 and lymph node status, there were no differences in OS or DFS rates between APE1 low and high-level expression groups. However, within the Ki-67 low-level expression cases, APE1 high-level expression was significantly associated with a better OS but not DFS rate. There were some patients lost to follow-up whose recurrence was unknown but whose death was confirmed. Also among patients followed up until recurrence or death, patients in high APE1 expression group showed that first recurrence site was mainly soft tissue, breast and bone. These might cause the difference between DFS and OS associated with APE1 expression. Within the high Ki-67 cases, APE1 low-level expression was correlated with a better OS rate, though statistical significance was not reached. This may indicate that in low-proliferating tumor cells, low-level nuclear APE1 expression still plays a role in carcinogenesis because of a reduction in its DNA repair function or a decreased ability to activate transcription factors.

## Conclusions

This study suggests that APE1 expression may be associated with prognosis in breast cancer. In particular, its role as a prognostic factor could be significant for breast cancers with a low proliferation index. It is proposed that nuclear APE1 may be a novel target in breast cancer with a low proliferation rate to obtain better outcome.
